# Metabolic Syndrome in Psoriasis Is Associated With Upregulation of CXCL16 on Monocytes and a Dysbalance in Innate Lymphoid Cells

**DOI:** 10.3389/fimmu.2022.916701

**Published:** 2022-06-17

**Authors:** Lisa Schielke, Nick Zimmermann, Sarah Hobelsberger, Julian Steininger, Anne Strunk, Kristin Blau, Jessica Hernandez, Stephan Künzel, Robert Ziegenbalg, Sarah Rösing, Stefan Beissert, Susanne Abraham, Claudia Günther

**Affiliations:** Department of Dermatology, University Hospital, Technical University Dresden, Dresden, Germany

**Keywords:** psoriasis, CXCL16, ILC, metabolic syndrome, obesity, monocytes, CXCR6

## Abstract

Psoriasis is frequently associated with the metabolic syndrome and occurs more often in obese individuals. In order to understand innate immune mechanisms mediating this inflammatory pattern we investigated expression of the chemokine and lipid scavenger receptor CXCL16 in patients with psoriasis and associated comorbidities. CXCL16 expression was enhanced on all monocyte subsets in psoriatic patients compared with healthy controls and positively correlated with psoriasis activity and severity index, body mass index and the risk for cardiovascular disease indicated by PROCAM score. The intensity of CXCL16 expression on monocytes further correlated with their capability to phagocytose oxidized LDL indicating the possibility to transform into foam cells in atherosclerotic plaques. Patients with psoriasis and atherosclerosis or obesity displayed elevated numbers of innate lymphoid cells in blood with specific increase of the IFN-γ or IL-17 producing ILC1 and ILC3 subpopulations. The expression of the CXCL16 receptor, CXCR6, was increased in ILCs and co-expressed with CCR6 but not CCR7 indicating their migratory potential to psoriatic skin or adipose tissue that is characterized by strong CXCL16 and CCL20 expression. This hypothesis was supported by the finding that the percentage of CXCR6 expressing ILCs was alleviated in blood of psoriatic patients. Together these data link a strong expression of CXCL16 to metabolic syndrome in psoriasis and indicate a possible link to ILC activation and tissue distribution in obese psoriatic patients. These data contribute to the understanding of the complex interaction of innate immunity and metabolic state in psoriasis.

## Introduction

Psoriasis vulgaris is a chronic inflammatory disease affecting approximately 2-3% of the population with pathognomonic chronic scaling erythematous plaques at the skin (1). However, psoriasis does not only involve the skin but is now regarded as systemic disease because psoriasis patients have an increased prevalence of metabolic syndrome (2–5). In 20% to 50% psoriasis is associated with hyperlipidemia, obesity, hypertension or peripheral insulin resistance (6–8) causing an elevated cardiovascular risk (CVR) and premature mortality (4, 9–11). Obesity has also been described as an independent risk factor for the development of psoriasis (12). Consequently, reasons for the association between metabolic disorders and psoriasis vulgaris are currently explored. In particular, the innate immune system is suggested to promote metabolic disorders and initiate cutaneous manifestations of psoriasis vulgaris. Innate lymphoid cells (ILCs) represent an important component of the innate immune system. They arise from a common lymphoid progenitor and are located in mucosal barriers and several tissues, including the intestine, lung, skin and adipose tissue (13–15). ILCs are divided into three different subsets – ILC1, ILC2, and ILC3 – according to their dependence on distinct lineage-determining transcription factors and cytokine secretion profiles (16). Group 1 ILC include the natural killer cells (NK) and the non-killer-cell ILC1. They are defined by their capability to produce amounts of IFN-γ but also TNF-α, IL-1β and IL-6 (17, 18). Group 2 ILCs are able to produce TH2 cell associated cytokines, including IL-4, IL-5, IL-9 and IL-13 (19). The Group 3 ILC are competent producers of the TH17 cell-associate cytokines IL-17 and IL-22 and have been described in psoriatic skin (15, 20, 21). Circulating and adipose tissue resident ILC1 have been more frequently detected in obese individuals resulting in aggravating morbidities like insulin resistance and metabolic disorders (22–24). In addition, Pantelyushin et al. demonstrated that mice lacking NK cells and ILCs did not develop psoriasiform plaques after topically administered imiquimod in contrast to normal mice, indicating a substantial impact of ILCs in the skin inflammation of psoriasis vulgaris (25). The role of human ILCs in obese individuals concerning the initiation and aggravation of psoriasis vulgaris is not well understood.

Mobilization from bone marrow and distribution of ILCs in different organs is orchestrated by chemokines. Innate lymphoid cell precursors can be recruited from bone marrow due to expression of CXCR6. The corresponding chemokine CXCL16 is circulating in blood and upregulated in psoriatic skin (26). The chemokine is unique as it is one of only two chemokines that is expressed as a membrane bound protein exerting its chemotactic function after enzymatic cleavage (27–29). We have shown that early innate immune stimulation by TLR ligands can induce CXCL16 expression in keratinocytes and monocytes (30). CXCL16 contributes to psoriatic inflammation by mediating recruitment of neutrophils and CXCR6+ CD8+ T cells into human skin (26). Interestingly, membrane bound CXCL16 on peripheral monocytes also acts as a scavenger receptor for oxidized low-density lipoproteins (oxLDL) (31). The phagocytosis and accumulation of lipids in monocytes or macrophages can accelerate the conversion into foam cells and promote lipid plaques in arterial vessels (32–35), that suggests a link between cutaneous inflammation and associated comorbidities in psoriasis.

Therefore, we investigated the expression of CXCL16 on proinflammatory monocytes and its role in lipid uptake in psoriasis associated atherosclerosis and metabolic syndrome.

## Results

### CXCL16 Expression on Monocytes Correlates With Metabolic and Cutaneous Disease Activity in Psoriasis

To investigate the role of the innate immune system in metabolic inflammation in psoriasis we examined CXCL16 expression on monocytes in 49 patients with psoriasis (mean age 57 ± 16.8 years, 33% female; mean PASI 18 ± 9.3) and correlated these levels with the extend of skin involvement and associated comorbidities. Hypertension and obesity (BMI ≥ 30 kg/m^2^) were present in 74% and 53% of the patients, respectively (**Table 1**). 37% of the patients presented with peripheral insulin resistance (pre-existing diabetes treated by oral antidiabetic drugs or insulin or elevated HbA1c), 43% had a hyperlipidemia (high serum level of cholesterol, HDL, LDL or triglyceride) and 47% were diagnosed with steatosis or atherosclerosis. 13 patients met all criteria for metabolic syndrome (27%) including insulin resistance, hyperlipidemia, obesity, and hypertension. One study participant aged 20 years had no other disorders besides psoriasis vulgaris.

**Table 1:** Metabolic comorbidities in the investigated patients.

Comorbidity	Number and Percentage of Total Patients (n=49)	Mean Age[years]	Percentage of Female Patients [%]
Peripheral insulin resistance	n= 18; 37%	63.9 ± 12.5	39
hypertension	n= 36; 74%	59.5 ± 16.5	25
obesity	n= 26; 53%	58.3 ± 15.6	42
hyperlipidemia	n= 21; 43%	59.7 ± 16.8	38
steatosis	n= 23; 47%	54.7 ± 15.6	30
atherosclerosis	n= 23; 47%	61.9 ± 14.8	39

**Figure 1:**
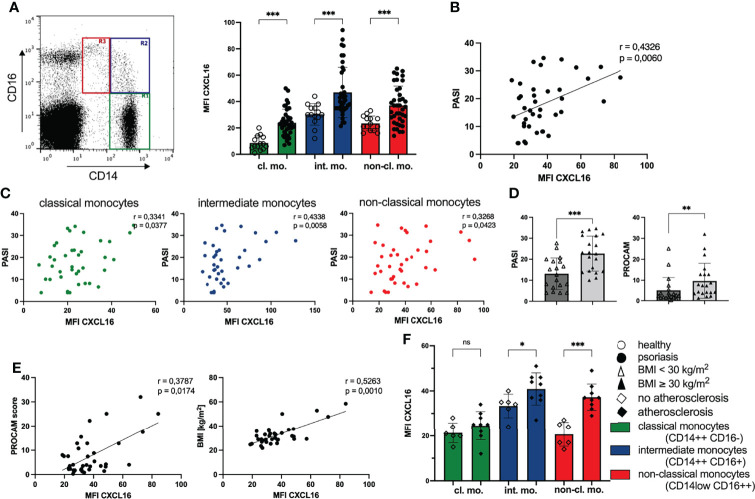
Expression of CXCL16 on monocytes is associated with enhanced clinical disease activity and cardiovascular risk. **(A)** Flow cytometry analysis of CXCL16 expression on monocyte subpopulations defined by gating for CD14 and CD16 expression in PBMC: classical monocytes (CD14++ CD16-, R1 green), intermediate monocytes (CD14++ CD16+, R2 blue) and non-classical monocytes (CD14low CD16++, R3, red), mean and SD of healthy controls (n= 14; unfilled dots) and patients with psoriasis vulgaris (n= 39; filled dots), Welch’s t test. **(B)** Correlation of CXCL16 expression on all monocytes of psoriatic patients (n=39) and clinical disease activity (PASI), Pearson’s correlation. **(C)** Correlation for each monocyte subset defined by color indicated in **(A)** and PASI score, Pearson’s correlation. **(D)** Mean and SD of PASI (unpaired t test) and PROCAM (Mann-Whitney test) of normal-weight (unfilled triangles; n=19) and obese psoriatic patients (filled triangles; n=20). **(E)** Correlation of chemokine expression with cardiovascular risk (PROCAM score); and body-mass-index (BMI). Each dot represents an individual patient, Pearson correlation. **(F)** CXCL16 expression on monocytes in psoriatic patients without atherosclerosis (n= 6; unfilled trapezoid) or with atherosclerosis (n= 9; filled trapezoid), mean and SD, unpaired t test. *p < 0.05; **p < 0.01; ***< 0.001. ns, not significant.

We found an enhanced expression of CXCL16 on all three monocyte subpopulations divided into classical monocytes with high expression of CD14 and low CD16 (CD14++CD16-), intermediate monocytes with expression of both antigens (CD14++CD16+) and the non-classical monocytes characterized by high amounts of CD16 and low CD14 on their surface (CD14low CD16++) (36) compared with healthy individuals (**Figure 1A**). In particular, the expression of CXCL16 on classical monocytes was increased threefold compared with controls (mean MFI CXCL16 healthy: 8.6; mean MFI CXCL16 psoriasis: 23.9). The highest expression of membrane bound CXCL16 was detected on intermediate monocytes of psoriatic patients (mean MFI CXCL16: 46.9) (**Figure 1A**). Interestingly, the intensity of CXCL16 expression on all monocytes and the monocytes subsets correlated significantly with the skin involvement scored by psoriasis activity and severity index (PASI) (**Figures 1B, C**). Obese patients with psoriasis showed a more severe clinical skin activity as well as a higher cardiovascular risk compared with non-obese patients (**Figure 1D**).

Furthermore, the expression of CXCL16 on peripheral monocytes of psoriatic patients correlated significantly with PROCAM (Prospective Cardiovascular Münster Study) score (**Figure 1E**) that indicates patients’ risk for developing cardiac diseases, such as myocardial infarction or cardiac death in the next 10 years (37). Extending this analysis, we searched for further correlations of chemokine expression with parameters potentiating the cardiovascular risk. We found a positive correlation of CXCL16 expression on monocytes and the body-mass-index in 39 psoriatic patients (**Figure 1E**). In addition, psoriatic patients with diagnosed atherosclerosis by medical imaging (transabdominal ultrasound or computed axial tomography or thoracal X-ray) displayed higher amounts of CXCL16 on their peripheral blood monocytes than psoriatic patients without atherosclerosis (**Figure 1F**). The clearest difference between these two groups was found in the expression of CXCL16 on non-classical monocytes with a nearly twice elevated chemokine expression in patients with psoriasis and atherosclerosis (MFI CXCL16 no atherosclerosis: 20.6; MFI CXCL16 atherosclerosis: 37.2). However, we did not observe a correlation between CXCL16 expression on monocytes and serum glucose, HbA1c or serum lipids (data not shown).

### Expression of CXCL16 on Monocytes Correlates with Their Capacity to Take Up oxLDL

**Figure 2:**
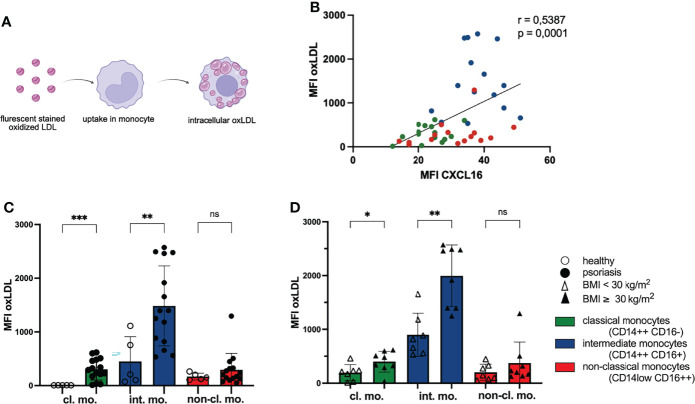
Phagocytosis of oxLDL by monocytes increases with CXCL16 expression. **(A)** PBMC were incubated with red fluorescing oxLDL for 3 h and subsequently stained for CD14, CD16 and CXCL16 expression. **(B)** The mean fluorescence intensity (MFI) of CXCL16 and oxLDL is shown. Every psoriasis patient is represented by three dots (n= 15). Colors represent monocyte subsets as defined in **Figure 1A**, Spearman correlation. **(C)** oxLDL concentrations in monocytes subsets of healthy controls (unfilled dots; n=5) and patients with psoriasis (filled dots; n=15), mean and SD, unpaired t test. **(D)** intracellular oxLDL concentrations in monocytes of normal-weight psoriatic patients (unfilled triangles; n=7) and obese patients (filled triangles; n=8), mean and SD, unpaired t test. *p < 0.05; **p < 0.01; ***p < 0.001. ns, not significant.

The membrane bound chemokine CXCL16 on monocytes is also a scavenger receptor for oxLDL (31). This promoted us to analyze the phagocytosis of oxLDL by CXCL16 on monocytes. We incubated PBMC with red fluorescing oxLDL for 3 hours and explored the fluorescence intensity of membrane bound CXCL16 on monocytes and intracellular oxLDL (**Figure 2A**). We found a positive correlation of the expressed membrane bound chemokine CXCL16 on the cell surface of monocytes and their uptake of oxLDL indicating that monocytes with elevated levels of CXCL16 on their surface accumulated phagocytosed oxLDL (**Figure 2B**). In line with this correlation, the classical and intermediate monocytes of psoriatic patients took up significantly more oxLDL than the monocytes of healthy individuals (**Figure 2C**). The mean fluorescence intensity of oxLDL in classical monocytes of psoriatic patients was 304 in comparison to classical monocytes of healthy volunteers with a MFI of 6. Likewise, the MFI of oxLDL in intermediate monocytes was increased threefold in psoriatic patients (MFI oxLDL healthy: 450; MFI oxLDL psoriasis: 1484). Interestingly, intermediate monocytes expressing highest amounts of CXCL16 on their surface also accumulated most intracellular oxLDL and monocytes of obese psoriatic patients accumulated a higher amount of oxLDL compared with normal-weight patients (**Figure 2D**).

The uptake of oxLDL by monocytes/macrophages is an important pathogenic step in the development of atherosclerosis and occurs in arterial vessel walls (38). The migration of monocytes into this area might be supported by CXCL16 because the chemokine is also expressed by endothelial cells (34). Staining CXCR6 on monocyte subpopulations revealed highest expression on intermediate monocytes (**Supplementary Figure 1**). Interestingly, this subgroup had the highest capacity of oxLDL phagocytosis implicating a potential role in atherosclerotic plaque formation.

### Metabolic Syndrome in Psoriasis Is Associated With an Upregulation of ILCs

**Figure 3:**
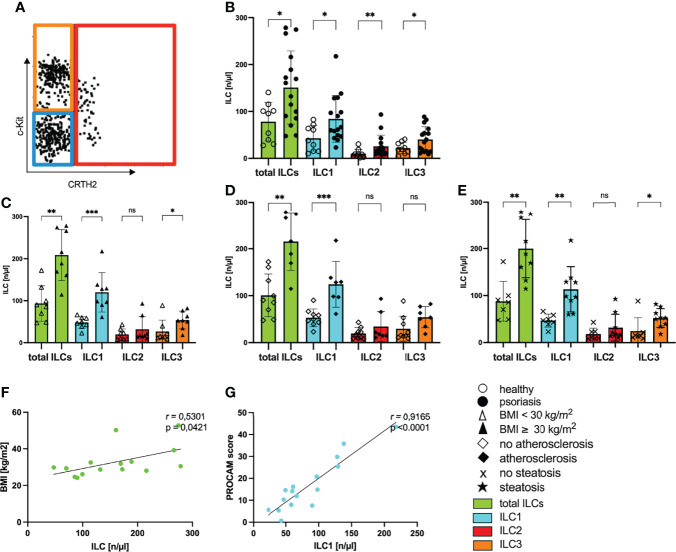
Increased numbers of ILCs in blood of psoriatic patients with metabolic disorders. **(A)** Gating strategy to define subpopulations of innate lymphoid cells ILC1, ILC2, ILC3. Analysis was performed by flow cytometry by using lineage Cocktail-Pacific Blue (Biolegend), CD11c-BV421, CD34-BV421, CD94-BV421, FcERIa-BV421, CD45-VioGreen, CD117-PE (c-Kit), IL7RA-APC (CD127), CRTH2-APC-Cy7 (CD294). ILCs are pregated as CD45 positive cells that are further defined by negative expression of the lineage cocktail and positive staining for IL7RA (**Supplementary Figure 2**). The figure is presenting cells covering these criteria and their further subdivision by individual expression of surface antigens CD117 and CRTH2. ILC1 are blue bordered (CD117-, CRTH2-), ILC2 red (CRTH2+) and ILC3 orange (CD117+, CRTH2-). Total ILCs are colored in green. **(B)** Number of ILCs of healthy controls (unfilled dots) and patients with psoriasis vulgaris (filled dots) in blood, mean and SD, each dot represents an individual patient, Mann-Whitney test. **(C)** Number of ILCs in blood of normal-weight psoriatic patients (body mass index, BMI<30 kg/m^2^; unfilled triangle; n=8) and obese patients (BMI ≥30 kg/m^2^; filled triangle; n=8) are shown, mean and SD, Mann-Whitney test. **(D)** Number of ILCs in blood of psoriatic patients without atherosclerosis (unfilled trapezoid; n= 9) and with atherosclerosis (filled trapezoid; n=7) are shown, mean and SD, Mann-Whitney test. **(E)** Number of ILCs of psoriatic patients without hepatic remodeling (cross symbol; n=7) and patients with steatosis hepatis (star; n=9), mean and SD, Mann-Whitney test. **(F)** Linear correlation of the number of circulating ILC and BMI (n=15), Pearson correlation. **(G)** Linear correlation of the number of circulating ILC1 and PROCAM score [%] (n=16), Pearson correlation. *p < 0.05; **p < 0.01; ***p < 0.001. ns, not significant.

Obesity and metabolic diseases are associated with a dysbalance in innate lymphoid cells (39) that have also been suggested to play an important role in cutaneous psoriatic inflammation (21, 40–42). To investigate a possible systemic role for ILCs in the metabolic disorder psoriasis we first analyzed the composition of ILCs in the blood of psoriasis patients. Upon multicolor fluorescence staining and gating (**Figure 3A**) we observed enhanced levels of total ILCs and all three subgroups of ILCs in psoriatic patients compared to healthy controls (**Figure 3B**) indicating a general activation in patients with psoriasis. Each ILC subset was nearly twofold enhanced in psoriatic patients (mean ILC1 healthy: 42.8/µL and psoriasis: 84.1/µL; mean ILC2 healthy: 9.9/µL and psoriasis: 25.8/µL; mean ILC3 healthy: 21.9/µL and psoriasis: 40.33/µL) and the population of ILC1 was most frequent (**Figure 3B**).

When we examined the number of ILCs in psoriatic patients with metabolic disorders, we found significantly increased levels of ILCs in obese patients with psoriasis compared with non-obese patients (**Figure 3C**). Among the subsets, ILC1 were nearly twofold increased in patients with psoriasis and obesity (**Figure 3C**) indicating that there is a rise in the amount of ILC1 in psoriatic patients with metabolic disorders. In line with these results, the number of total ILCs and ILC1 were also enhanced in patients with additional hepatic steatosis or atherosclerosis (**Figures 3D, E**). The number of total ILCs correlated positively with the BMI of psoriatic patients (**Figure 3F**). Underlining the impact of ILC1 in psoriatic patients with metabolic disorders, we found a positive correlation between the number of circulating ILC1 and the PROCAM score measuring the cardiovascular risk (**Figure 3G**).

**Figure 4:**
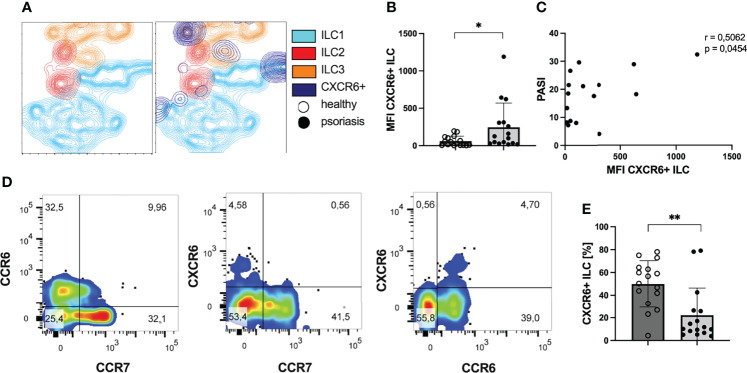
ILCs express CXCR6 in patients with psoriasis. **(A)** T-SNE contour plot presenting the clustering of ILCs in subgroups ILC1 (blue), ILC2 (red) and ILC3 (orange) and their expression of the chemokine receptor CXCR6 (dark blue). There are subgroups of ILC1 and ILC3 expressing the receptor CXCR6. **(B)** Flow cytometry analysis of MFI of CXCR6 expression on peripheral ILCs of psoriatic patients (n=16) and healthy volunteers (n=16), mean and SD, Welch t test. **(C)** Correlation of MFI of the receptor CXCR6 and the clinical skin involvment (n= 16), Pearson correlation. **(D)** Coexpression of chemokine receptors CXCR6, CCR6 and CCR7 among all ILCs. **(E)** Mean and SD of percentage of CXCR6 expressing ILCs among all ILCs of healthy volunteers (n=16) and psoriatic patients (n=16), Mann-Whitney test. *p < 0.05; **p < 0.01.

ILCs develop from innate lymphoid precursor cells which express high levels of CXCR6 (43), the chemokines receptor for CXCL16. CXCR6 was expressed on a subset of ILC subpopulation with the highest proportion in ILC1 and ILC3 where we observed well-demarcated CXCR6 positive subpopulations among ILC1 and ILC3 (**Figure 4A**). The intensity of CXCR6 expression on ILCs was enhanced in psoriatic patients (**Figure 4B**). In addition, mean expression intensity of CXCR6 on ILCs correlated with the PASI score indicating an association of skin involvement with the intensity of CXCR6 expression (**Figure 4C**). CXCR6 was only found in CCR7 negative ILCs and all of CXCR6+ ILCs co-expressed CCR6 (**Figure 4D**). This pattern indicated that CXCR6 expressing ILCs might be recruited to peripheral organs and this recruitment might be supported by CCR6. Indeed, when we analyzed the percentage of peripheral ILCs expressing the receptor CXCR6 on their surface, the numbers were reduced in patients with psoriasis (**Figure 4E**). This finding suggests that CXCR6+ ILCs are not prone to CCL21 or CCL19 mediated migration to lymphoid tissue (44) but rather migrate to peripheral organs with high CXCL16 expression such as the skin (26, 28) or atherosclerotic plaques (35) and that this pathway is active in obese individuals with elevated CXCL16 expression.

## Discussion

Here we show that the metabolic disease state in psoriasis is associated with an upregulation of the chemokine and lipid receptor CXCL16 on monocytes of psoriatic patients and the activation of ILCs. Expression of CXCL16 was increased on all subsets of monocytes circulating in the blood of psoriatic patients and this expression correlated not only with the PASI score, indicating severity of cutaneous involvement, but also with the amount of adipose tissue, as indicated by an elevated BMI.

It is known that obesity and increased body mass index (BMI) caused by high calorie diet are significant risk factors for the development of psoriasis (45–47). High calorie diet may exacerbate or trigger disease (46). Adipose tissue functions as endocrine organ that plays a key role in lipid and glucose metabolism, insulin-mediated processes and inflammation. The systemic effects are mediated by large abdominal white adipose tissue (48). However, adipose tissue in the skin might also impact the local inflammation (48). Secretion of cytokines such as IFN-γ and TNF-α by adipose tissue macrophages (49) could stimulate the expression of proinflammatory chemokines like CXCL16 in monocytes and keratinocytes in the skin (50). Further studies have shown that a high fat western diet is associated with an increased expression of type I interferon (IFN) and IFN stimulated genes (51). We have previously demonstrated, that type I IFNs are strong stimuli of CXCL16 upregulation in psoriatic dermis and epidermis that could support the cutaneous migration of neutrophils and CD8+ T cells (26, 52). Recently, expression of CXCR6 has been described on slan positive monocytes that correspond to the CD14low CD16++ non-classical monocytes (53). The cells migrated *in vitro* to CXCL16 which might support their recruitment into the skin of psoriatic patients where they accumulate and contribute to perpetuation of the disease (53–55).

On monocytes, CXCL16 is not only a chemokine but also a scavenger receptor for oxidized lipids (31). Interestingly, a high expression of CXCL16 on monocytes was associated with a stronger uptake of oxLDL which might have implications for the evolution of systemic disease manifestations in psoriasis. Lipid-overloaded monocytes may convert into foam cells in the intima (56) forming lipid plaques and arterial stenosis (32, 34). In addition, patients with metabolic syndrome reveal a higher percentage of circulating CXCR6 expressing platelets and CXCR6 expressing platelet-bound neutrophils, resulting in enhanced CXCR6/CXCL16-dependent adhesion to the dysfunctional arterial endothelium (57). Attached platelets form a white thrombus and present continuously CXCR6 to further circulating CXCL16 positive peripheral blood mononuclear cells (PBMCs) enhancing inflammation (58). Furthermore, endothelial CXCL16 is acting as potent adhesion ligand (58). This could be relevant for platelets, neutrophils and also for the immigration of monocytes that partly express CXCR6. (53). Therefore, chemokine CXLC16 und chemokine receptor CXCR6 are potent adhesion molecules in the atherosclerosis-prone vessel walls and thus may promote the progression of atherosclerosis. In line with this, psoriatic patients with coincident atherosclerosis expressed elevated levels of CXCL16 on monocytes compared with patients without vascular comorbidities. These findings suggest that CXCL16 expression on monocytes contributes to the development of cardiovascular diseases in psoriatic patients, which is further supported by the observed correlation between CXCL16 expression on monocytes and an enhanced PROCAM score.

In addition to the scavenger receptor function of CXCL16 that might result in lipid plaque formation, CXCL16 may induce cellular proliferation of aortic smooth muscle cells and pro-inflammatory cytokines in an NF-kappa B-dependent manner (33) and aggravate inflammation by adhesion of CXCR6 positive immune cells (34).

Among the innate immune cells perpetuating atherosclerosis are ILC1 (59). Here, we demonstrate that ILCs and especially ILC1 cells are more frequent in blood of psoriasis patients and the highest numbers are found in patients with atherosclerosis or obesity. ILC1 are characterized by their capability to produce high amounts of IFN-γ but also TNF-α, IL-1β and IL-6 (17, 18). Beside the role of these cytokines in psoriatic inflammation, ILC1 derived IFN-γ can accelerate atherosclerosis in mice (59). In addition, IFN-γ can affect macrophage homeostasis in the visceral adipose tissue by polarization toward M1 macrophages and the preferential killing of adipose M2 macrophages (22, 60). The proinflammatory M1 macrophages are capable to secrete TNF-α and IL-6 which may lead to insulin resistance (61, 62) and CXCL16 upregulation (30, 34).

Part of ILC1 expressed the chemokine receptor CXCR6 that might be relevant for their recruitment into atherosclerotic plaques, adipose tissue and even the skin. The expression of CXCR6 on ILCs was elevated in psoriasis patients. CXCR6 was expressed on a well described population of CCR7 negative ILCs. As CCR7 is required for guidance to lymphoid tissues by CCL19 and CCL21 (44, 63), the CXCR6 expressing cells might reflect a population on its way to peripheral organs with high CXCL16 expression such as the skin (28), atherosclerotic plaques (35) or the adipose tissue (64). Nearly all CXCR6+ ILCs co-expressed CCR6, the chemokine receptor for CCL20 which is known to induce migration of CCR6 expressing T cells to the skin (65) and is also upregulated in adipose tissue of obese individuals (66). This co-expression might enhance the migratory potential of the cells. The pattern was found in ILC1 and ILC3. The latter are of special importance for the cutaneous inflammation in psoriasis because they are competent producers of the TH17 cell-associated cytokines IL-17 and IL-22 (15, 20).

The finding demonstrating a reduced percentage of CXCR6 expressing ILCs in blood of patients with psoriasis compared with healthy controls might further support the concept of an active recruitment of CXCR6+ cells to peripheral sites. Trapping of ILCs in skin, atherosclerotic plaques or adipose tissue can explain their alleviation in the peripheral blood.

Following the current development in ILC differentiation and clustering that is driven by single cells sequencing a recent publication described CD94 positive cytotoxic ILCs upregulated in Crohn´s disease (67). As Crohn´s disease is associated with psoriasis (68) it would be interesting to investigate the prevalence of this population in psoriasis in a future analysis. Here, CD94+ cells were excluded as part of the NK-cell exclusion strategy following Soare et al. (69).

In conclusion, we showed that CXCL16 expression could be an important link between metabolic syndrome, the elevated cardiovascular risk and skin involvement of psoriasis vulgaris. Obesity was associated with an elevated expression of CXCL16 on monocytes that promoted the phagocytosis of oxLDL by scavenger receptor function of CXCL16. OxLDL loaded monocytes can contribute to the development of atherosclerosis. Secretion of CXCL16 in skin and atherosclerotic plaques might support accumulation of proinflammatory ILCs as innate immune stimulators that promote psoriatic inflammation. Therefore, therapeutic strategies could aim in reducing the expression of CXCL16 in psoriatic patients to prevent systemic manifestations. Collado et al. showed that CXCL16 neutralization by the angiotensin-1-receptor antagonist losartan significantly inhibited cell adhesion to arterial vessels. This effect was mediated by downregulation of CXCL16 expression on endothelial cells and therefore CXCL16 inhibition may positively affect the risk of metabolic disorders in psoriatic patients (57).

## Materials and Methods

### Patients

Blood samples of 49 patients with psoriasis were collected for further analysis of CXCL16 expression, oxLDL phagocytosis and determination of ILC subsets. The mean age of all patients was 57 ± 16.8 years, 33% female, mean PASI 18 ± 9.3. In most patients diagnosis of psoriasis has been known since 18 ± 16.2 years. In 23 of these patients, clinical data on serum lipids, HbA1c, CRP, blood pressure and medical imaging like transabdominal sonography or X-ray were assessed. The patients did not receive any systemic treatment with immunosuppressive drugs for at least 6 months. Diagnosis of psoriasis was confirmed by clinical and histologic criteria. Blood from age-matched healthy volunteers (mean age 58 ± 17,9 years, 68% female) was used as control. The investigational protocols (EK251062016) were approved by the Ethics Committee of the University Hospital of the Technical University Dresden according to the Declaration of Helsinki.

### Flow Cytometry Staining of Monocytes and Analysis of oxLDL Uptake

PBMCs were isolated from heparinized whole blood of patients with psoriasis and healthy controls by density gradient centrifugation using Biocoll separating solution (Biochrom, Berlin, Deutschland). Contaminating serum components were removed by washing the cells with PBS (phosphate buffered saline, containing 2% fetal calf serum, 0.02% NaN3) and cells were incubated with sort buffer (phosphate buffered saline, containing 2% fetal calf serum) for 10 minutes to block unspecific bindings. In order to assess low-density lipoprotein uptake, 1x10^6^ cells/ml or 200.000 cells/200µl were seeded in 96 v-bottom well plate in RPMI-1640 medium (Gibco LifeTechnologies, Carlsbad, CA, containing 10% fetal calf serum, 2 mM L-glutamine, 1% nonessential amino acids, 100 U/ml penicillin, and 100 mg/ml streptomycin). Thereafter, 10µg/ml human Dil-labeled medium oxidized low-density lipoprotein (Dil-oxLDL, Kalen Biomedical, Germantown, USA #770232-9) was added and the cells incubated for at least 3 hours at 37°C. Subsequently, the cells were washed with PBS and cells were stained for CXCL16, CD14 and CD16 expression with fluorescence labeled antibodies (**Table 2**). The incubation with antibodies was performed for 30 minutes in the dark at 4°C on ice and followed by washing in PBS. Dead cells were excluded by Propidium Iodide (0,5 µg/ml) that was added directly before flow cytometry analysis was performed on a five-laser/18channel system (LSR Fortessa, Becton Dickenson, Franklin Lakes, NJ). Determination of transmembrane CXCL16 and intracellular oxLDL was analyzed with FlowJo software. Gating of cell populations were defined by isotype controls. Monocytes were characterized by expression of their surface antigens CD14 and CD16. Classical monocytes: CD14++CD16-, intermediate monocytes: CD14++CD16+ and non-classical monocytes: CD14low CD16++.

**Table 2:** Antibodies used for flow cytometry immunofluorescence for PBMC staining.

Antibody	Manufacturer/Catalogue No.
CD14 -APC	BD Bioscience, San Jose, USA #555399
CD16 -PerCP	BioLegend, San Diego, USA #302029
CXCL16 -FITC	R&D Systems, Abingdon, UK #FAB976G
normal goat IgG isotype -FITC	R&D Systems, Abingdon, UK #Ab-108-C

### Flow Cytometry Staining of Innate Lymphoid Cells Subsets

Whole blood (20ml) from healthy controls and psoriatic patients was collected into EDTA tubes. First, 500 μl of whole blood were put into one tube and mixed with fiuorochrome-labeled antibodies (**Table 3**) or respective isotype controls following previous reports (69) and incubated for 20 minutes. After washing, erythrocytes (RBCs) were lysed and nucleated cells were fixed using RBC lysis and fixation solution (BioLegend, Fell, Germany). Subsequently, flow cytometry analysis was performed on a five-laser/18channel system (LSR Fortessa, Becton Dickenson, Franklin Lakes, NJ) and analyzed with FlowJo software. ILCs were defined as CD45+, IL7RA+, negative for lineage markers CD3, CD11c, CD14, CD16, CD19, CD20, CD34, CD56, CD94, FcERIa (**Supplementary Figure 2**). In addition, ILC1 were defined as CRTH2-CD117-fraction, ILC2s as CRTH2+ CD117-, and ILC3s as CRTH2- CD117+ fractions, respectively (**Supplementary Figure 2**). Absolute numbers of cells were calculated as following: % ILC x absolute lymphocyte count (automated count) = absolute number of ILC per cubic millimeter. For calculation, the absolute lymphocyte count was determined by routine automated complete blood count measurement.

**Table 3:** Antibodies used for flow cytometry immunofluorescence for ILC staining.

Antibody	Manufacturer/Catalogue No.
lineage cocktail (CD3, CD14, CD16, CD19, CD20, CD56) – Pacific Blue	BioLegend, San Diego, USA #348805
CD11c -BV421	BioLegend, San Diego, USA #301628
CD34 -BV421	BioLegend, San Diego, USA #343610
CD94 -BV421	BD Bioscience, San Jose, USA #743948
fcERIa – BV421	BioLegend, San Diego, USA #334624
CD45 -VioGreen	Miltenyi, Bergisch Gladbach, Germany #130-113-124
CD127 (IL7RA) -APC	BioLegend, San Diego, USA #351316
CD117 -PE	BioLegend, San Diego, USA #313204
CD294 (CRTH2) -APC	BioLegend, San Diego, USA #350114
CXCR6 -BV737	BD Bioscience, San Jose, USA #748449
mouse IgG2a isotype -BV737	BD Bioscience, San Jose, USA #612765
mouse IgG2a isotype -BV650	BioLegend, San Diego, USA #400266
mouse IgG2b isotype -PerCP	BioLegend, San Diego, USA #400338

### Analysis of Chemokine Receptor Expression on ILCs

For analysis of multiple chemokine receptor expression on ILCs, staining was performed on PBMC to increase the cell number. PBMC were separated from whole blood by density gradient centrifugation using Biocoll separating solution (Biochrom, Berlin, Deutschland). After isolation, cells were incubated with sort buffer (phosphate buffered saline, containing 2% fetal calf serum) for 10 minutes to block unspecific bindings. Then, cells were fixed in formaldehyde 4% and stained with the antibodies listed in **Table 3**. Additionally, mouse anti-human IgG against CCR7 -BV650 (BioLegend, #353234) and mouse anti-human IgG against CCR6 – PerCP (BioLegend, #353406) were added. Flow cytometry analysis was performed on a five-laser/18channel system (LSR Fortessa, Becton Dickenson, Franklin Lakes, NJ). Staining was analyzed with FlowJo software.

### Statistical Analysis

Data are presented as bars (indicating mean ± standard deviation) or dot plots (indicating the correlation coefficient r and the two-tailed (two-sided) P value performed by correlations). Test for normal distribution was performed by Shapiro-Wilk test. In normally distributed values unpaired t test possibly with Welch’s correction or Pearson correlation were used. For nonparametric tests Mann-Whitney test and Spearman correlation were used. Unless otherwise indicated computation with the help of GraphPad Prism 10 (GraphPad Software, San Diego, CA). In all cases, *P < 0.05 was considered to be statistically significant (**P < 0.01, ***P < 0.001).

## Data Availability Statement

The raw data supporting the conclusions of this article will be made available by the authors, without undue reservation.

## Ethics Statement

The studies involving human participants were reviewed and approved by Ethics Committee of the University Hospital of the Technical University Dresden. The patients/participants provided their written informed consent to participate in this study.

## Author Contributions

CG conceived and directed the project. NZ and SR designed and LS performed experiments. LS and CG wrote the manuscript. SH, JS, AS, SK, and KB were involved in the recruitment and diagnosis of psoriasis patients. All authors listed have made a contribution to the work and approved the submitted version.

## Funding

This study received funding from Novartis and Pfizer and Deutsche Forschungsgemeinschaft (German Research Foundation), grant TRR237 369799452/404458960 to CG. The funders were not involved in the study design, collection, analysis, interpretation of data, the writing of this article or the decision to submit it for publication.

## Conflict of Interest

The authors declare that the research was conducted in the absence of any commercial or financial relationships that could be construed as a potential conflict of interest.

## Publisher’s Note

All claims expressed in this article are solely those of the authors and do not necessarily represent those of their affiliated organizations, or those of the publisher, the editors and the reviewers. Any product that may be evaluated in this article, or claim that may be made by its manufacturer, is not guaranteed or endorsed by the publisher.
