# Supplementary material for: Metabolic Syndrome in Psoriasis Is Associated With Upregulation of CXCL16 on Monocytes and a Dysbalance in Innate Lymphoid Cells
Source: Front Immunol. 2022 Jun 17;13:916701. doi: 10.3389/fimmu.2022.916701 (PMC9248801; doi:10.3389/fimmu.2022.916701)
Supplement: Supplementary file 1 [file DataSheet_1.pdf]

Supplementary Material

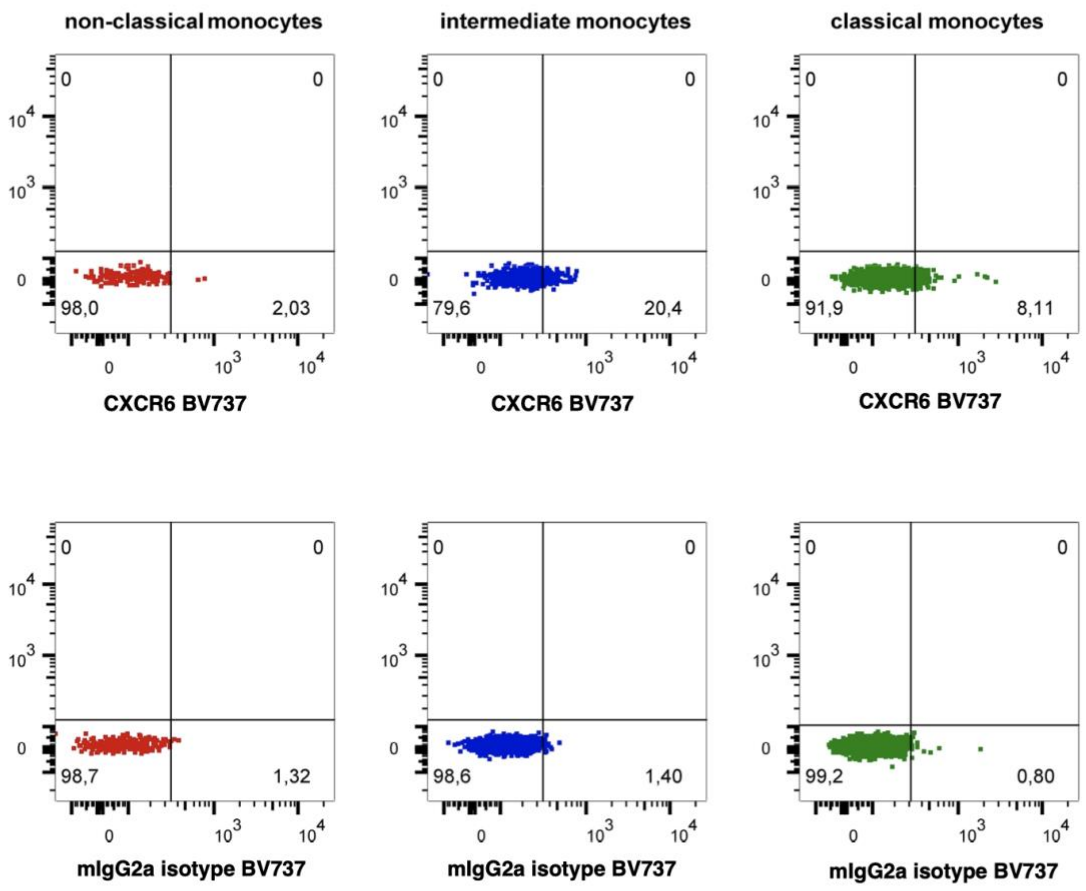

Supplementary Figure 1. Expression of CXCR6 by monocyte subsets

Flow cytometry analysis of CXCR6 expression and staining of isotype control on monocyte subpopulations defined by gating for CD14 and CD16 expression in PBMC: non-classical monocytes (CD14low CD16++, R3, red), intermediate monocytes (CD14++ CD16+, R2 blue) and classical monocytes (CD14++ CD16-, R1 green). Percentages of CXCR6 positive and negative cells are shown. Especially the intermediate monocytes express the chemokine receptor CXCR6.

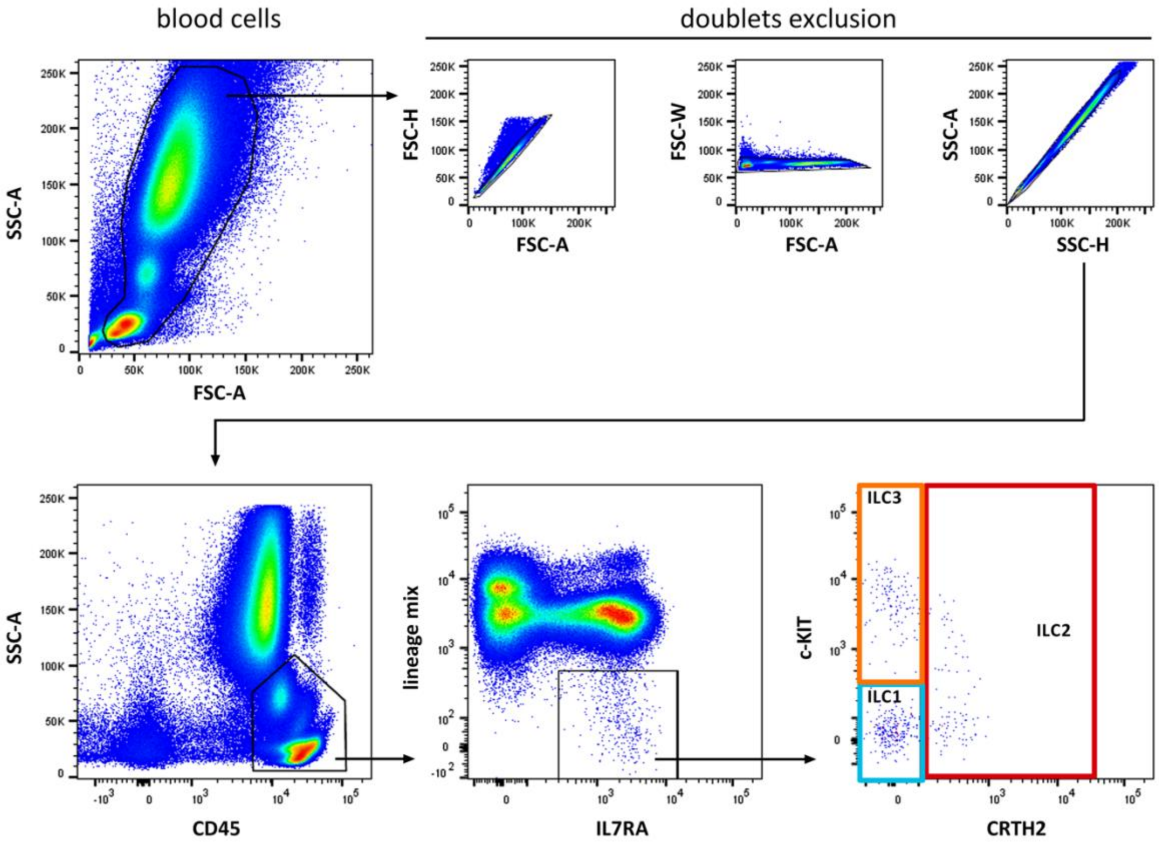

Supplementary Figure 2. Gating strategy to define subpopulations of innate lymphoid cells ILC1, ILC2, ILC3

Gating was first performed in forward and sideward scatter and doublets were excluded as indicated. CD45 was used to identify the ILC containing cell population that was further subdivided in lineage (CD3, CD11c, CD14, CD16, CD19, CD20, CD34, CD56, CD94, FcERIa) negative and IL7RA positive cells. Further subtyping was performed by individual expression of surface antigens CD117 (c-KIT) and CRTH2. ILC1 are blue bordered (CD117-, CRTH2-), ILC2 red (CRTH2+) and ILC3 orange (CD117+, CRTH2-).
